# Results and lessons from dual extraction of DNA and RNA from formalin-fixed paraffin-embedded breast tumor tissues for a large Cancer epidemiologic study

**DOI:** 10.1186/s12864-022-08837-6

**Published:** 2022-08-25

**Authors:** Rochelle Payne Ondracek, Jianhong Chen, Beth Marosy, Sirinapa Szewczyk, Leonard Medico, Amrutha Sherly Mohan, Priya Nair, Rachel Pratt, Janise M. Roh, Thaer Khoury, John Carpten, Lawrence H. Kushi, Julie R. Palmer, Kim Doheny, Warren Davis, Michael J. Higgins, Song Yao, Christine B. Ambrosone

**Affiliations:** 1 Department of Cancer Prevention and Control, Roswell Park Comprehensive Cancer Center, Elm & Carlton Streets, Buffalo, NY 14263 USA; 2 Center for Inherited Disease Research, Johns Hopkins Genomics, Institute of Genetic Medicine, Johns Hopkins School of Medicine, Baltimore, MD USA; 3 Department of Molecular and Cellular Biology, Roswell Park Comprehensive Cancer Center, Buffalo, NY USA; 4 Division of Research, Kaiser Permanente Northern California, Oakland, CA USA; 5 Department of Pathology, Roswell Park Comprehensive Cancer Center, Buffalo, NY USA; 6 Department of Translational Genomics, Keck School of Medicine, University of Southern California, Los Angeles, CA USA; 7 Slone Epidemiology Center, Boston University, Boston, MA USA

**Keywords:** FFPE, Population study, Nucleic acids extraction, Breast tumor

## Abstract

**Background:**

The use of archived formalin-fixed paraffin-embedded (FFPE) tumor tissues has become a common practice in clinical and epidemiologic genetic research. Simultaneous extraction of DNA and RNA from FFPE tissues is appealing but can be practically challenging. Here we report our results and lessons learned from processing FFPE breast tumor tissues for a large epidemiologic study.

**Methods:**

Qiagen AllPrep DNA/RNA FFPE kit was adapted for dual extraction using tissue punches or sections from breast tumor tissues. The yield was quantified using Qubit and fragmentation analysis by Agilent Bioanalyzer. A subset of the DNA samples were used for genome-wide DNA methylation assays and RNA samples for sequencing. The QC metrices and performance of the assays were analyzed with pre-analytical variables.

**Results:**

A total of 1859 FFPE breast tumor tissues were processed. We found it critical to adjust proteinase K digestion time based on tissue volume to achieve balanced yields of DNA and RNA. Tissue punches taken from tumor-enriched regions provided the most reliable output. A median of 1475 ng DNA and 1786 ng RNA per sample was generated. The median DNA integrity number (DIN) was 3.8 and median DV200 for RNA was 33.2. Of 1294 DNA samples used in DNA methylation assays, 97% passed quality check by qPCR and 92% generated data deemed high quality. Of the 130 RNA samples with DV200 ≥ 20% used in RNA-sequencing, all but 5 generated usable transcriptomic data with a mapping rate ≥ 60%.

**Conclusions:**

Dual DNA/RNA purification using Qiagen AllPrep FFPE extraction protocol is feasible for clinical and epidemiologic studies. We recommend tissue punches as a reliable source material and fine tuning of proteinase K digestion time based on tissue volume.

**Impact:**

Our protocol and recommendations may be adapted by future studies for successful extraction of archived tumor tissues.

**Supplementary Information:**

The online version contains supplementary material available at 10.1186/s12864-022-08837-6.

## Background

The extraction of high-yield and high-quality DNA and RNA from a limited amount of tumor tissue is a critical first step in tissue-based cancer genomic research. While fresh frozen (FF) tissues are preferred for the yield of nucleic acids with better quality, they may not be available from most patients due to logistic challenges in obtaining and processing them in a timely manner. Alternatively, formalin-fixed paraffin-embedded tissue (FFPE) tumor tissues are more widely available, even from patients diagnosed decades earlier. Because of the tissue fixing step and the long storage time, the damage and fragmentation of nucleic acids in archived FFPE samples were a concern for sample quality for downstream genetic profiling. However, technological advances in recent years have made it possible to use DNA and RNA samples derived from FFPE tissues for sequencing, microarray hybridization, and other applications [1–5].

Many nucleic acid extraction kits are commercially available, some specialized for either DNA or RNA, with a few being compatible for DNA and RNA dual extraction from the same input tissues. Several previous studies have evaluated and compared their performance for FFPE tumor tissues [6–10]. One study showed success with FFPE tissue stored for up to 40 years [6], and another study concluded that the Qiagen AllPrep dual extraction kit had the best performance for FFPE tissues [10]. Because the goals of those prior studies were to identify an appropriate method for extractions, they were often conducted in a small number of samples using multiple kits simultaneously for comparison. It remains to be seen how these extraction methods perform in “real-world” projects where hundreds to thousands of tissues need to be processed.

In comparison to DNA- or RNA-only extraction, a DNA and RNA dual extraction method is more appealing than single nucleic acid extraction, for reasons of more efficient use of the precious tumor tissues and the fact that DNA and RNA derived from the same bulk tissues will allow for more coherent multi-omics analysis.

We recently completed dual DNA and RNA extraction from more than 1800 FFPE breast tumor tissues for a large epidemiologic project. We selected the Qiagen AllPrep DNA/RNA FFPE kit based on the literature [10] and our preliminary work. However, given the varying quality of FFPE samples, we found it critical to evaluate each batch of tissues before extraction and fine-tune the laboratory protocol from batch to batch accordingly. Different from previous reports that conducted comparisons of different extraction approaches, we present here a summary of the results and lessons we learned from our large-scale practice, which might be useful for future tissue-based studies. To this end, we provide details on the laboratory procedures, quantification and QC measures of the generated nucleic acid samples, and the impact of pre-analytical variables on the performance of the extraction work.

## Results

A total of 1859 dual DNA/RNA extractions were completed using FFPE breast tumor punches and sections. Table 1 summarizes the yield and QC matrices of the resultant DNA and RNA samples. We generated a median of 1475 ng (range 0–20,636 ng) DNA, with a median DIN of 3.8 (range 1–31), and a median of 1786 ng (range 0–31,753) RNA, with a median RIN of 1.4 (range 1–5.6) and a median DV200 of 33.2 (range 1–98.5). RT-PCR assays both in presence (+) or absence (−) of reverse transcription reactions with 10 RNA samples, including 5 samples of ~ 30 μg yield (high end level) and 5 samples of ~ 2.5 μg yield (average level), revealed no DNA contamination in RNA samples generated from this dual extraction protocol.

**Table 1:** Summary of the yield and quality of DNA and RNA samples extracted from FFPE breast tumor tissues

	DNA			RNA			
	n	Median Yield (Range) (ng)	Median DIN (Range)	n	Median Yield (Range) (ng)	Median RIN (Range)	Median DV200 (Range)
All samples	1859	1475 (0–20,636)	3.8 (1–31)	1859	1786 (0–31,753)	1.4 (1–5.6)	33.2 (1–98.5)
Tissue age							
≤ 5 years	162	2241 (0–18,480)	4.9 (1–6.6)	162	1058 (0–24,816)	1.4 (1–2.9)	38.5 (9–72)
5–6 years	287	1602 (0–17,248)	4.2 (1–6.3)	287	1213 (0–18,800)	1.4 (1–3.2)	39.6 (8.7–72.4)
7–8 years	363	1586 (0–16,324)	3.7 (1–5.9)	363	1626 (0–16,732)	1.4 (1–2.7)	36.1 (5.9–70)
9–11 years	436	1497 (0–20,636)	3.9 (1–6.2)	436	2228 (0–25,192)	1.4 (1–2.6)	35.2 (1–98.5)
12–14 years	307	1602 (0–15,277)	3.5 (1–6.1)	307	2310 (0–19,928)	1.4 (1–5.6)	28.3 (4.1–94.3)
15+ years	304	744 (0–10,749)	2.9 (1–31)	304	2792 (0–31,753)	1.4 (1–3.9)	25.3 (2.9–83.2)
*p*-value for trend		*p* < 0.001	*p* < 0.001		*p* < 0.001	p = 0.26	*p* < 0.001
Tissue type							
punch	1629	1602 (0–20,636)	3.9 (1–31)	1629	1861 (0–31,753)	1.4 (1–5.6)	34.9 (1–98.5)
section	230	482 (0–13,891)	3 (1–6.6)	230	1640 (0–14,907)	1.4 (1–3.9)	22.2 (2.9–66.8)
*p*-value		*p* = 0.002	*p* < 0.001		*p* = 0.004	*p* = 0.12	*p* < 0.001
Procedure type							
excision	1815	1512 (0–20,636)	3.8 (1–31)	1815	1861 (0–31,753)	1.4 (1–5.6)	33.4 (1–98.5)
core biopsy	44	179 (0–6068)	3 (1–6.6)	44	638 (0–12,220)	1.5 (1–2.4)	24.1 (6.1–66.8)
*p*-value		*p* < 0.001	*p* = 0.01		*p* < 0.001	*p* = 0.58	*p* = 0.65

The yield of DNA trended lower as the tissue aged (trend *p* < 0.001) (Table 1). In contrast, the yield of RNA trended higher as tissue aged (trend *p* < 0.001). Both DIN and DV200 were lower in older tissues (DIN: trend *p* < 0.001; DV200: trend *p* < 0.001), yet no difference was found for RIN by tissue age (trend *p* = 0.26). The yield and quality of both DNA and RNA was better from punches than from sections (Table 1), yet the relationships of DNA/RNA yields and quality with tissue age remained consistent between punches and sections (Supplementary Tables S1 and S2). The procedure type from which the tissues were originally obtained also had an impact on the extraction results. Those from excisions had higher DNA and RNA yield and better quality than those from core biopsy, although the difference in DV200 was not statistically significant (Table 1).

**Fig. 1:**
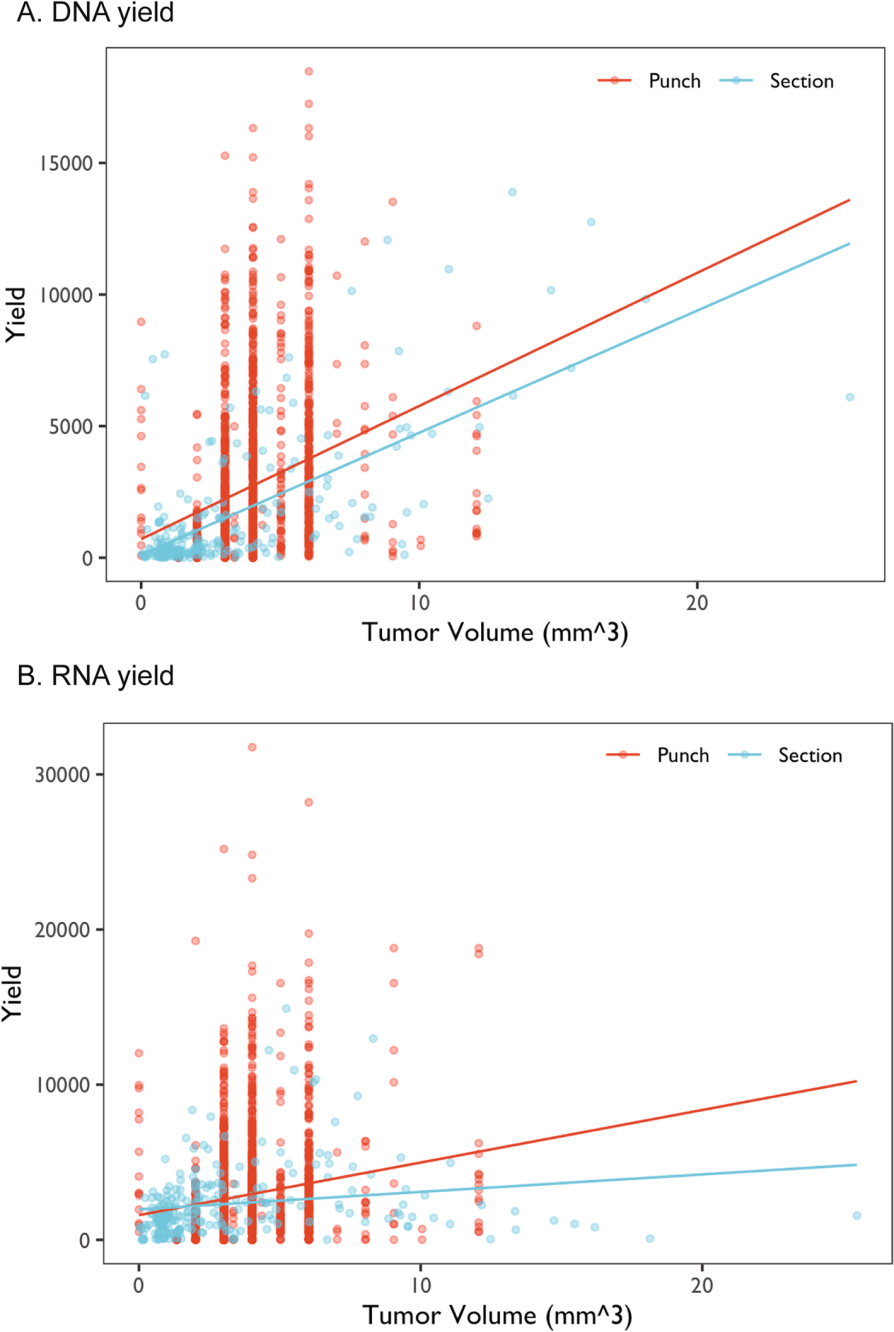
Correlation plots of the yields of DNA and RNA samples with the estimated tumor volume

**Fig. 2:**
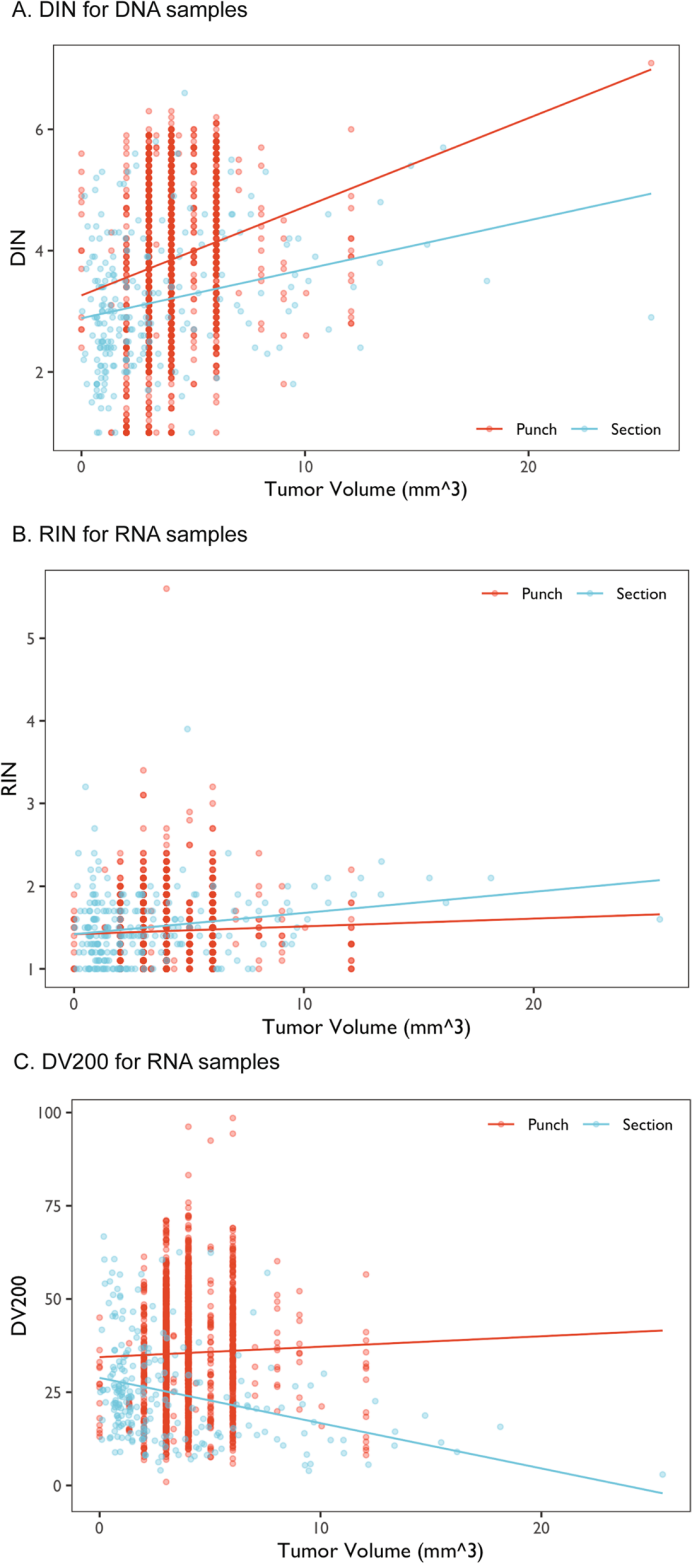
Correlation plots of the quality measures of DNA and RNA samples with the estimated tumor volume

The relationships between tissue volume and DNA and RNA yields are shown in Fig. 1. As expected, higher tissue volume produced higher yields. This relationship was evident for both tissue punches and sections. The relationships between tissue volume and DIN, RIN and DV200 are shown in Fig. 2. Higher tumor volume generally predicted better quality measures of DNA samples (Fig. 2a), yet there appears to be no correlation between tissue volume and RNA samples derived from tissue punches. Interestingly, for tissue sections, a larger tissue volume was correlated with slightly higher RIN (Fig. 2b) but significantly lower DV200 (Fig. 2c).

qPCR results as a part of QC check by CIDR before DNA methylation microarray assays were available from 1294 of the DNA samples. As shown in Table 2, of all the DNA samples evaluated, only 3% fell into the worst quality bin of 5 that might predict a failure in subsequent methylation profiling. There was a weak correlation between older tissue age and worse quality score (trend *p* < 0.001).

**Table 2:** Summary of qPCR results of tumor DNA samples used for methylation microarray assays

	All Samples	qPCR CT Bin Category				
		1 (Best Quality)	2	3	4	5 (Worst Quality)
Count (precent)	1294 (100)	600 (46)	383 (30)	190 (15)	84 (6)	37 (3)
Tissue age						
≤ 5 years	134 (10)	96 (16)	24 (6)	12 (6)	2 (2)	0 (0)
5–6 years	214 (17)	120 (20)	55 (14)	30 (16)	7 (8)	2 (5)
7–8 years	267 (21)	127 (21)	81 (21)	39 (21)	16 (19)	4 (11)
9–11 years	305 (24)	148 (25)	95 (25)	35 (18)	19 (23)	8 (22)
12–14 years	203 (16)	47 (8)	73 (19)	46 (24)	23 (27)	14 (38)
15+ years	171 (13)	62 (10)	55 (14)	28 (15)	17 (20)	9 (24)
*p*-value for trend	*p* < 0.001					
Tissue type						
punch	1154 (89)	520 (87)	350 (91)	177 (93)	77 (92)	30 (81)
section	140 (11)	80 (13)	33 (9)	13 (7)	7 (8)	7 (19)
*p*-value	*p* = 0.23					
Procedure type						
excision	1279 (99)	594 (99)	376 (98)	190 (100)	83 (99)	36 (97)
core biopsy	15 (1)	6 (1)	7 (2)	0 (0)	1 (1)	1 (3)
*p*-value	*p* = 0.52					

Table 3 summarizes the performance of the 1294 DNA samples profiled using the MethylationEPIC assay. 92% of the samples yielded high-quality methylation data, 4% yielded data of marginal quality, whereas the other 4% failed to yield usable data. When tissue age was considered, 69% of those that failed to generate usable data were at least 12 years old; yet, of all the 374 tissues of such age attempted, 305 (82%) still yield high-quality data and 40 (11%) failed. No significant association was found between sample performance and tissue type or procedure type.

**Table 3:** Summary of the performance of DNA methylation assays

	All Samples	Assay Performance		
		High quality	Marginal	Unusable
Count (precent)	1294 (100)	1190 (92)	46 (4)	58 (4)
Tissue age				
≤ 5 years	134 (10)	132 (11)	2 (4)	0 (0)
5–6 years	214 (17)	210 (18)	3 (7)	1 (2)
7–8 years	267 (21)	257 (22)	2 (4)	8 (14)
9–11 years	305 (24)	286 (24)	10 (22)	9 (16)
12–14 years	203 (16)	172 (14)	10 (22)	21 (36)
15+ years	171 (13)	133 (11)	19 (41)	19 (33)
*p*-value	< 0.001			
Tissue type				
punch	1154 (89)	1064 (89)	36 (78)	54 (93)
section	140 (11)	126 (11)	10 (22)	4 (7)
*p*-value	0.19			
Procedure type				
excision	1279 (99)	1177 (99)	46 (100)	56 (97)
core biopsy	15 (1)	13 (1)	0 (0)	2 (3)
*p*-value	0.22			

A subset of 341 tumor DNA samples were used for whole-exome sequencing (WES). Sequencing libraries generated from all samples attempted passed QC. The average number of total reads generated for each sample was 398 million (range 65–1511 million). The average reads map rate was 0.880 (range 0.178–0.999). The average rate of PCR duplicate was 0.216 (range 0.004–0.690). The average sequencing depth was 203x (range 21x-672x). An average of 86.7% bases had a minimum 20x coverage (range: 33.2–99.6%), with 27 of 341 (7.9%) samples below a minimum cutoff of 80%.

Another subset of 130 tumor RNA samples were used for RNA sequencing, which generated an average of 69.2 million 100-bp paired-end reads. The average mapping rate (a proportion of uniquely mapped reads out of all input reads) was 82.3% (range: 22.6–95.5%), with only 5 samples below 60%. The average alignment rate (the percentage of mapped reads being assigned to a gene feature) was 65.4% (range: 12.6–87.2%). There was a weak correlation between mapping rate and DV200 (Pearson r = 0.29, *p* = 0.0007).

## Discussion

Our data from processing 1859 FFPE breast tumor tissues demonstrated that dual extraction of DNA and RNA samples from the same input materials for a large epidemiologic study is feasible. We were able to generate a large quantity of DNA and RNA samples with satisfactory quality from most tissues attempted. The results showed that tissue punches taken from tumor-enriched regions provided the most reliable source materials with higher yields and quality of nucleic acids and from dual extraction. This could be due to the small surface area of the tissue punches exposed to the atmosphere, which can protect and slows down nucleic acid degradation in cells packed inside the punches.

For dual DNA/RNA extraction, it is critical to strike a balance between the yields of DNA and RNA. Based on our experience, the proteinase K digestion time is the most important factor to control in order to achieve a desirable balance. We thus fine-tuned this step based on the estimated tissue volume and grouped those with similar volume in the same batches, which allowed us to adjust the proteinase K digestion time from batch to batch.

Tissue age tends to be a concern for nucleic acid extraction from FFPE tissues. Most of the tissues in our study were more than 5 years old, and a significant proportion were more than 10 years old. Although nucleic acid fragmentation tended to become worse in older tissues, the extent of the degradation appeared to be acceptable even after more than 10 years since the tissues were archived. In fact, 82% of DNA samples derived from tissues of 12+ year old in our study yielded high quality methylation data. We also observed that as FFPE tissues aged, the yields of DNA trended down as expected, but surprisingly, the yields of RNA trended up. This might again reflect the balance between the DNA and RNA yields. We did not adjust the proteinase K digestion time based on tissue age, which might warrant testing in future work.

Although we obtained good results from tissue sections of 10-μm or thicker, we refrained from using tissue sections thinner than 10-μm for dual extraction. In our pilot testing, the RNA quality from 5-μm sections tended to be worse than those from RNA-only extraction using the same input material. In fact, this can be seen in the negative correlation between the estimated tissue volume and DV200 of RNA samples derived from tissue sections (Fig. 2c), which might be due to a larger surface area exposed to the atmosphere and thus worse fragmentation. Interestingly, an opposite trend was found for RIN, which is another measure of RNA fragmentation. For downstream applications such as RNA-sequencing, DV200 is more widely used as a quality measure.

A limitation of our study was our work was conducted entirely with breast tumor tissues. Caveats and additional optimization work might be necessary when applying the method to other tissue types.

## Conclusions

In conclusion, dual DNA/RNA extraction using an optimized Qiagen AllPrep FFEP kit is feasible for clinical and epidemiologic studies where a large number of archived tumor tissues, including those stored more than a decade ago, need to be processed into DNA and RNA for downstream molecular analysis. We recommend tissue punches as a reliable source material and also recommend fine tuning of the proteinase K digestion time based on tumor tissue volume available for extraction.

## Materials and methods

### Processing of the archived tissue specimens

FFPE tumor tissues processed in this work came from three breast cancer studies, including the Women’s Circle of Health (WCHS), the Black Women’s Health Study (BWHS), and the Pathways Study (Pathways). Details on study design, patient recruitment, and data and biospecimen collection have been described previously [11–14]. WCHS is a case-control study designed to investigate risk factors for aggressive breast cancer in Black and White women [11–13]. BWHS is a prospective cohort study among Black women, with an emphasis on disease development, especially cancer [13]. Pathways is a prospective study of breast cancer survivorship at Kaiser Permanente in Northern California [14]. Participants in the three studies gave written informed consent for their archived tumor specimens to be obtained from clinical laboratories and used for research related to breast cancer. The present project was approved by the institutional review boards of Roswell Park Cancer Institute, Boston University Medical Campus, and Kaiser Permanente Northern California.

All tissues were received and processed at the Data Bank and BioRepository (DBBR) laboratories at Roswell Park Comprehensive Cancer Center, following an established workflow as detailed below. Tissue samples in the form of FFPE blocks were preferred when available; otherwise, unstained slides were requested. As part of the routine tissue processing for tumor blocks, hematoxylin and eosin (H&E) slides were created from the blocks and reviewed by a board-certified breast pathologist to identify tumor areas. If the tumor area was large enough, two 14-gauge punches (1.6 mm diameter, 6 mm thickness) were taken; otherwise, only one 14-gauge punch was taken. The tissue punches were stored in -80 °C until the time of use. When unstained section slides were received in lieu of blocks, 20- or 10-μm sections were used for nucleotide extraction. After pathological review of H&E slides to identify tumor areas, the non-tumor tissues and extra paraffin were macro-dissected away as much as possible before the tumor tissues were scrapped off the slides.

### Optimization of the nucleic acid extraction protocol

The Qiagen AllPrep DNA/RNA FFPE kit was chosen for dual DNA and RNA extraction based on the available literature and our hands-on experience with the method in pilot work. The kit allows for simultaneous purification of total RNA and genomic DNA from the same input FFPE tissue by optimizing the lysis step, a key step leading to differentially release of RNA and DNA. Two types of tissue samples, including punches and sections, were used. To optimize the performance of the extraction protocol, several special measures both before and during the extraction procedures were taken. First, for both sample types, the assessment of tumor tissue size was done before extraction. For tissue punches, the relative length of a core containing tumor but not paraffin was estimated; for tissue sections, the total area size of tumor regions as circled by the pathologist was estimated based on the digitalized H&E images. This assessment allowed the grouping of tissues with similar size so the later lysis step could be fine-tuned based on the tumor size. Second, tissue punches were spread as thin as possible by using a manual compressor, to maximize the tissue area for better proteinase K digestion. For tissue sections, as many as 5 slides were used (fewer sections were used for larger tumors). Third, the most important optimizing step was the lysis time by proteinase K, which controls the amount of RNA released into supernatant and the amount of DNA that remained in tumor tissue. An approximately equal final yield of RNA and DNA was attempted by fine tuning the lysis time based on the estimated tumor size. The manufacturer’s protocol recommends 15 minutes; whereas 3 ~ 5 minutes were used in our extraction, which achieved balanced yields of the two nucleic acids.

### Tissue digestion

Except for the above optimization steps, we generally followed the manufacturer’s protocol. Tissue flakes were placed in a 1.5 ml sterilized Eppendorf tube followed by deparaffinization using 1 ml xylene for 2 minutes. Xylene was removed after centrifugation at full speed (20,816 x *g*) for 2 minutes and the pellet was washed by adding 1 ml 100% ethanol to the pellet. After centrifuge at full speed for another 2 min, supernatant was removed, and the pellet was dried by keeping the lid open at room temperature for 10 min. The pellets were resuspended in 150 μl Buffer PKD evenly followed by adding 10 μl proteinase K and mixed thoroughly. Depending upon tissue size, the reaction was incubated at 56 °C for 3–5 minutes. The reactions were then stopped by immediately transferring the tubes to ice for 3 minutes, followed by centrifuge at full speed for 15 minutes. Supernatants were transferred to a new 1.5 ml RNase free Eppendorf tube without disturbing the pellets for RNA purification. The pellets were saved for DNA purification. From this step on, experiments were split into RNA and DNA extraction.

### RNA extraction

The supernatants were incubated at 80 °C for exact 15 minutes. Before applying the supernatants to a RNeasy MinElute spin column, 320 μl Buffer RLT and 1120 μl 100% ethanol were added into sample and mixed thoroughly to adjust the column binding condition. The mixture was loaded onto a RNeasy MinElute spin column followed by centrifugation at 8000 x *g* for 15 s. To equilibrate the DNase I digestion reaction, 350 μl Buffer FRN was applied to the column followed by centrifugation at 8000 x *g* for 15 s. Nucleic acid molecules were now bound to column and ready for the DNase I digestion. 80 μl DNase I incubation solution (10 μl DNase I stock solution mixed with 70 μl Buffer RDD gently) was directly pipetted to the center of the RNeasy MinElute spin column membrane and placed on the benchtop at room temperature for 15 minutes. After the digestion reaction, 500 μl Buffer FRN was applied to the RNeasy MinElute spin column. Flow-through was saved after a short spin 8000 x *g* for 15 s and applied to the same spin column in a new 2 ml collection tube. The column was washed by adding Buffer RPE (twice, each using 500 μl) and RNA column was washed further with 500 μl of 100% ethanol. The RNeasy MinElute spin column was placed in a new 2 ml collection tube and centrifuge at full speed for 5 minutes to remove any residual ethanol on columns. To elute RNA, 50 μl RNase-free water was added directly to the center of the spin column membrane and allowed to sit at room temperature for 3 minutes, followed by centrifuge at full speed for 2 minutes. 2 ul of RNA samples were used for QC and the remainder were stored in -80 °C.

### DNA extraction

Pellets saved for DNA extraction were resuspended in 180 μl Buffer ATL, added 40 μl proteinase K, and mixed by vertexing. A second proteinase K digestion was carried out in water bath at 56 °C overnight. To reverse crosslinking, reactions were incubated at 90 °C for 2 hours without disturbing. Before transferring to a QIAamp MinElute spin column, 200 μl Buffer AL and 200 μl of 100% ethanol were added sequentially and mixed thoroughly by vertexing or pipetting. The mixtures were loaded onto the column by centrifuge at 8000 x *g* for 15 s. The column was washed sequentially by adding 700 μl Buffers AW1, AW2, and 100% ethanol. At last, the QIAamp MinElute spin column was centrifuged at full speed for 5 min to remove residual ethanol. DNA was eluted in 80 μl Buffer ATE by centrifuge at full speed for 2 minutes after sitting at room temperature for 3 minutes. 2 ul DNA samples were used for QC and the rest of the samples were stored in -80 °C.

### Nucleic acid quantification and QC

Nucleic acid quantification and QC were performed by the Roswell Park Genomics Shared Resource (GSR). The concentration of DNA and RNA was determined on a Qubit 3.0 Fluorometer (ThermoFisher Scientific), using the dsDNA HS (High Sensitivity) and RNA BR (Broad-Range) Assay kits, respectively. The size distribution of RNA fragments was assessed using RNA 6000 Nano Kit (Agilent) on a 2100 Bioanalyzer Lab-on-a-Chip platform (Agilent Technologies, USA), and expressed as the percentage of fragments greater than 200 base pairs (DV200).

### Methylation-specific qPCR DNA assay and Illumina MethylationEPIC assay

Quality assessment of the FFPE-derived DNA was determined prior to methylation assay using Illumina Infinium HD FFPE QC kit by the Center for Inherited Disease Research (CIDR) at Johns Hopkins Genomics (https://support.illumina.com). Samples were normalized to a final concentration of 1 ng/uL and the assay used 2 ng total input DNA according to the manufacturer’s protocol. Triplicates of each sample underwent quantitative PCR using the QuantStudio 6 Real-Time PCR system (ThermoFisher Scientific). Ct (threshhold cycle) of Real-Time PCR experiments will be referred to as Cq (quantification cycle) as a quantification value. Herein, the ΔCq value is defined as the “quality score” and average CT values across triplicates are compared against a quality standard to generate a quality score. Illumina recommends that a Quality score values ≤5 be utilized for optimal assay performance (https://support.illumina.com). Following the quality assessment, Illumina MethylationEPIC assay for all samples available was carried out by CIDR following the manufacturer’s protocol.

### RT (reverse transcription)-PCR assay

A subset of 10 tumor RNA samples were used to test possible DNA contamination in the extracted RNA samples by RT-PCR assay on QuantStudio™ 6 Flex Real-Time PCR System (ThermoFisher Scientific), using TagMan human GAPD (GAPDH) primers (https://www.thermofisher.com/order/catalog/product/4333764T) and standard amplification protocol.

### Whole-exome sequencing

A subset of 341 tumor DNA samples were used for whole-exome sequencing, using the Agilent SureSelect Human Whole Exome kit and sequenced by an Illumina NovaSeq 6000 sequencer with 2 × 150 bp reads, following the manufacturer’s protocols.

### RNA sequencing assay

A subset of 130 tumor RNA samples which had a minimum of DV200 of 20% were used for RNA sequencing. The sequencing library was generated using Agilent SureSelect XT HS2 RNA kit and the resultant libraries were sequenced on an Illumina NovaSeq sequencer with 100-bp paired-end reads, following the manufacturers’ protocols.

### Statistical analysis

Simple descriptive statistics were used to summarize the yields and QC measures of the DNA and RNA samples extracted. Correlation plots were generated to visualize linear relationships between two numeric variables of interest, with the significance tested by Pearson correlation test. Across group comparisons were conducted using Student t-test, ANOVA for numeric variables, or Chi-square test or Fisher’s exact test for categorical variables. All analyses were performed in R 4.1.1.

## Supplementary Information

**Additional file 1: Table S1.** Summary of the yield and quality of DNA and RNA samples extracted from FFPE breast tumor tissue sections. **Table S2.** Summary of the yield and quality of DNA and RNA samples extracted from FFPE breast tumor tissue punches.

## Data Availability

All data generated or analyzed during this study are included in this article. Raw data is not publicly available but can be shared from the corresponding author on reasonable request with permission of C.B. Ambrosone, J.R. Palmer, and K Doheny.

## Abbreviations

FFPE: Formalin-fixed paraffin-embedded
DIN: DNA integrity number
FF: Fresh frozen
CIDR: The Center for Inherited Disease Research
WCHS: Women’s Circle of Health
BWHS: The Black Women’s Health Study
DBBR: Data Bank and BioRepository laboratories at Roswell Park Comprehensive Cancer Center
BCRF: The Breast Cancer Research Foundation
