# Supplementary material for: Results and lessons from dual extraction of DNA and RNA from formalin-fixed paraffin-embedded breast tumor tissues for a large Cancer epidemiologic study
Source: BMC Genomics. 2022 Aug 25;23:614. doi: 10.1186/s12864-022-08837-6 (PMC9404650; doi:10.1186/s12864-022-08837-6)
Supplement: Supplementary file 1 — Additional file 1: Table S1. Summary of the yield and quality of DNA and RNA samples extracted from FFPE breast tumor tissue sections. Table S2. Summary of the yield and quality of DNA and RNA samples extracted from FFPE breast tumor tissue punches. [file 12864_2022_8837_MOESM1_ESM.docx]

Table S1. Summary of the yield and quality of DNA and RNA samples extracted from FFPE breast tumor tissue sections

|  | DNA | | | RNA | | | |
| --- | --- | --- | --- | --- | --- | --- | --- |
|  | n | Median Yield (Range) (ng) | Median DIN (Range) | n | Median Yield (Range) (ng) | Median RIN (Range) | Median DV200 (Range) |
| All samples | 230 | 482 (0-13891) | 3 (1-6.6) | 230 | 1589 (0-12972) | 1.4 (1-3.9) | 22.225 (2.9-66.8) |
| Tissue age |  |  |  |  |  |  |  |
| ≤5 years | 25 | 1137 (32-12751) | 4.6 (1.8-6.6) | 25 | 1611 (5-12220) | 1.4 (1-2.4) | 40.85 (9-66.8) |
| 5-6 years | 49 | 407 (0-10133) | 3.5 (1-5.5) | 49 | 1025 (0-8366) | 1.3 (1-2.4) | 26.76 (10.4-60.6) |
| 7-8 years | 48 | 658 (54-13891) | 2.9 (1.6-4.9) | 48 | 1495 (38-10340) | 1.45 (1-2.2) | 23.605 (7-57) |
| 9-11 years | 39 | 370 (0-10965) | 3 (1.6-5.6) | 39 | 1814 (0-7934) | 1.5 (1-2.2) | 17.54 (7.9-60.5) |
| 12-14 years | 20 | 290 (18-7731) | 2.5 (1-4) | 20 | 1481 (376-5640) | 1.5 (1.2-2.7) | 18.735 (4.1-62.4) |
| 15+ years | 49 | 382 (0-6838) | 2.4 (1-4.6) | 49 | 2388 (23-12972) | 1.55 (1-3.9) | 14.59 (2.9-36.8) |
| r (p-value) |  | -0.14 (p=0.03) | -0.48 (p<0.0001) |  | 0.07 (p=0.28) | 0.17 (p=0.01) | -0.44 (p<0.0001) |

Table S2. Summary of the yield and quality of DNA and RNA samples extracted from FFPE breast tumor tissue punches

|  | DNA | | | RNA | | | |
| --- | --- | --- | --- | --- | --- | --- | --- |
|  | n | Median Yield (Range) (ng) | Median DIN (Range) | n | Median Yield (Range) (ng) | Median RIN (Range) | Median DV200 (Range) |
| All samples | 1629 | 1602 (0-20636) | 3.9 (1-31) | 1629 | 1824 (0-39856) | 1.4 (1-5.6) | 34.93 (1-98.5) |
| Tissue age |  |  |  |  |  |  |  |
| ≤5 years | 137 | 2587 (0-18480) | 4.9 (1-6.3) | 137 | 949 (0-24816) | 1.4 (1-2.9) | 38.34 (10-72) |
| 5-6 years | 238 | 1786 (0-17248) | 4.3 (1-6.3) | 238 | 1236 (0-18800) | 1.5 (1-3.2) | 41.43 (8.7-72.4) |
| 7-8 years | 315 | 1679 (0-16324) | 3.8 (1-5.9) | 315 | 1626 (0-16732) | 1.4 (1-2.7) | 37.76 (5.9-70) |
| 9-11 years | 397 | 1648 (0-20636) | 4 (1-6.2) | 397 | 2294 (0-39856) | 1.4 (1-2.6) | 36.17 (1-98.5) |
| 12-14 years | 287 | 1879 (0-15277) | 3.6 (1-6.1) | 287 | 2406 (0-19928) | 1.4 (1-5.6) | 29.27 (7.2-94.3) |
| 15+ years | 255 | 838 (0-10749) | 3.1 (1-31) | 255 | 2801 (0-31753) | 1.4 (1-2.5) | 27.57 (6.1-83.2) |
| r (p-value) |  | -0.16 (p<0.0001) | -0.23 (p<0.0001) |  | 0.21 (p<0.0001) | 0.001 (p=0.97) | -0.29 (p<0.0001) |
